# The impact of paid family leave in the United States on birth outcomes and mortality in the first year of life

**DOI:** 10.1111/1475-6773.13288

**Published:** 2020-04-05

**Authors:** Diana Montoya‐Williams, Molly Passarella, Scott A. Lorch

**Affiliations:** ^1^ Division of Neonatology Children's Hospital of Philadelphia Philadelphia Pennsylvania

**Keywords:** infant mortality, low birthweight, maternity leave, preterm birth

## Abstract

**Objective:**

To evaluate the effect of paid family leave in California on statewide rates of preterm birth, low birthweight, postneonatal mortality, and overall infant mortality.

**Data Sources:**

Live birth and death certificates from all in‐hospital deliveries occurring in California (state exposed to the family leave policy) and two unexposed states (Missouri and Pennsylvania) from 1999 to 2008 (n = 6 164 203).

**Study Design:**

We used a difference‐in‐differences approach to compare rates of infant health outcomes before and after implementation of the 2004 policy in California with rates in two states without paid family leave policies. Prespecified stratified analyses examined whether policy response differed by maternal characteristics. Conditional regression models using comparisons matched on a mother's likelihood of living in California in the pre–family leave period were then employed as sensitivity analyses to confirm our findings.

**Data Collection/Extraction Methods:**

Probabilistic methods were used to match live birth records to maternal and newborn hospital records. Only singleton births were included. Dyads were excluded if the infant gestational age was <23 weeks or greater than 44 weeks or if the birthweight was an outlier.

**Principal Findings:**

Compared to the unexposed states, adjusted postneonatal mortality rates decreased by 12 percent in California after 2004 (aOR 0.88, 95% CI 0.80‐0.97). There were no significant effects on the other outcomes. There were no differences in the effect by race/ethnicity or insurance status except for increased odds of low birthweight among privately insured women in California after 2004. Point estimates in the propensity score–matched sensitivity models were similar to the results of the fully adjusted models for all four outcomes, but confidence intervals crossed one.

**Conclusions:**

Implementation of paid family leave policies in California was associated with a 12 percent reduction in postneonatal mortality after adjusting for maternal and neonatal factors.

**What This Study Adds**

- Paid family leave has been linked to reduced infant mortality, especially in European nations.
- The effect of paid family leave policies on infant mortality using US data alone has not been explored.
- Postneonatal mortality rates dropped by 12 percent in California after its state legislature enacted the first partial pay family leave policy in the nation.
- An increased odds of low birthweight births among privately insured women were also seen after implementation of this paid family leave policy.

## 1 INTRODUCTION

Despite modest improvements in the last 20 years, infant mortality rates (IMR) in the United States, long considered an important indicator of the health of a nation, have persistently been higher than economically matched peer countries.^1^ Preterm birth and low birthweight, conditions known to occur at higher rates in the United States than other peer countries, help explain a significant proportion of this country's IMR disadvantage.^1^ However, even when accounting for low birthweight and/or preterm infants, cross‐national comparisons continue to find higher than expected IMRs in the United States.^2^ Much of this disadvantage appears to arise from higher rates of postneonatal mortality (ie, death after the first 28 days of life), even among infants born clinically well with a normal birthweight.^2^

Several social determinants of health have been linked to an increased risk of these adverse outcomes among vulnerable women. In particular, maternal stressors such as shift work, high job stress, and high physical demands have been associated with increased risk of adverse outcomes among vulnerable infants.^3^ As a result, parental work leave has been explored as a strategy to reduce adverse infant health outcomes. There is now a substantial body of literature which has linked parental leave to improved infant health outcomes, including reduced infant hospitalizations, low birthweight, premature birth, and infant mortality, as well as improved breastfeeding duration.^4^, ^5^, ^6^, ^7^ However, the most robust evidence tying family leave and infant mortality comes from large international studies looking mainly at European countries. These data have shown that the strongest relationship exists between *paid* family leave and reduced postneonatal mortality.^6^, ^8^

Evidence that parental leave improves birth outcomes in the United States has been accrued mainly from studying the effect of the federal Family and Medical Leave Act (FMLA) of 1993, which offers protected *unpaid* leave for up to three months to eligible workers.^5^ The study of paid parental leave in this country is scarce and has relied on data from the few states that offer partial pay coverage during parental leave, mainly through temporary disability insurance programs. One study indicates that even partially paid leave in the United States is associated with a decreased risk of low birthweight infants, particularly among black women and unmarried women.^9^ However, the effect of paid leave programs on infant mortality in this country, where approach to social policies often differs from Europe, has not been explored.

In 2004, California became the first state in the United States to implement a paid family leave (PFL) program.^10^ This policy offers any new parent wishing to bond with a newborn or care for a sick family member up to six weeks of partially compensated leave.^11^ The policy offers coverage to mothers after pregnancy‐related temporary disability insurance ends and to new mothers who do not have pregnancy‐related disability insurance. It also provides coverage to new fathers and new parents of foster or adopted children, thus effectively expanding eligibility for paid family leave to a larger segment of the population. After enactment of the policy, the percentage of women in California on maternity leave at any given time more than doubled (from 5.4 percent to 11.8 percent), indicating uptake of the policy in the population.^12^

The 2004 Paid Family Leave program in California has been associated with improved rates of breastfeeding^10^, ^13^ and decreased hospitalizations for abusive head trauma.^14^ However, its effect on birth outcomes and risk of mortality in the first year of life is not known. Given ongoing national discussions regarding national parental leave programs^15^ and the recent passage of bills expanding family leave in several other states such as New York, New Jersey, and Washington,^16^ studying the effect of the first PFL program in the country at the time of its implementation is crucial to better understand how such policies impact outcomes in the United States specifically. This study aimed to assess the effect of the 2004 implementation of paid family leave in California on statewide rates of preterm birth, low birthweight, postneonatal mortality, and overall infant mortality. It also sought to determine whether the policy had a heterogeneous effect among women with different socioeconomic profiles, and in particular, among those who experience disparities in the outcomes of interest.

## 2 METHODS

### 2.1 Study cohort

We used a quasi‐experimental before and after design to compare infant health outcomes in California before and after passage of paid family leave legislation. California served as the exposed group, whereas Missouri and Pennsylvania served as two separate unexposed controls. These control states were chosen due to their data availability, large number of deliveries, and large diverse populations. The paid family leave policy was passed in California in 2002 but did not begin paying benefits to eligible parents until July 1, 2004.^17^ The pre–paid family leave (pre‐PFL) period was defined as all births occurring from January 1, 1999, to December 31, 2003, and the post–paid family leave (post‐PFL) period was defined as births occurring from January 1, 2005, to December 31, 2008. Births occurring in 2004 were excluded to allow for a roll‐out period.

Our infant health outcomes were preterm birth (live birth occurring prior to 37 weeks), low birthweight (infant weighing < 2500 g), postneonatal mortality (death occurring between 29 and 365 days of life), and overall infant mortality (death occurring at any point in the first year of life).

We analyzed live birth and death certificates linked to hospital deliveries occurring in all three states from 1999 to 2009. Using state vital statistics and administrative hospital discharge data, the linkage was done by the states' Departments of Health using probabilistic methods after we acquired IRB approval from each of these entities. More than 98 percent of all live birth records were able to be matched to maternal and newborn hospital records as previously described.^18^ Unmatched records had similar gestational age and racial/ethnic distributions to the matched records, but 80 percent of them were missing a birth hospital on the birth certificate, suggesting these infants were not delivered in a hospital. Only singleton births were included in this analysis given the increased risk of prematurity and low birthweight associated with multiple gestations. Birth records were also excluded if they had a gestational age less than 23 weeks or greater than 44 weeks, if the birthweight was less than 400 grams or greater than 8,000 grams, or if the birthweight was more than five standard deviations from the mean birthweight for the gestational age in the cohort to exclude outliers likely caused by incorrect data entry.^19^

### 2.2 Data analysis

We first examined sample characteristics. To estimate the effect of the PFL law on the infant health outcomes, we used a difference‐in‐differences (DID) statistical approach. This econometric technique is commonly used in quasi‐experimental studies to analyze the effects of policy or legislation by comparing data in areas that have enacted a policy with data from areas that have not enacted the policy.^20^, ^21^ This approach reduces bias and assists in determining causality because the calculated difference creates an estimate of the effect of a policy while adjusting for secular trends in each outcome and unobservable and potentially confounding variables that cannot be controlled for, but whose prevalence remains similar between exposed and unexposed states.^22^

To test the validity of this approach in our context, we examined whether trends in each outcome were statistically different between California and the other states in the pre‐PFL period (ie, 1999‐2003), as this would violate the parallel trends assumption necessary for DID models.^23^ Table S1 outlines the findings of pre‐existing trends in the main outcomes prior to the passage of PFL.^24^ All confidence intervals include 1, indicating that there were no statistically significant trends occurring prior to 2004 in each outcome studied.

To estimate the impact of PFL on each outcome in our study, we constructed models that included year indicator variables to account for secular changes that affect all women similarly across states as well as state indicator variables that account for unobserved confounding fixed effects in each state that stay constant over time. Models also included an interaction term between California and the overall post‐PFL period. The interaction term is the difference‐in‐differences estimate of the association between paid family leave and the outcome.

Table S2 depicts the demographic makeup of each of our states in the year California passed its PFL policy. Multivariable logistic regression models were constructed to adjust for state differences in individual‐ and state‐level characteristics associated with the main outcomes. The maternal characteristics we adjusted for were age, education, insurance status, race/ethnicity, obesity, and tobacco, alcohol, or drug use. Labor‐related variables included county unemployment rate and median household income in the census tract of maternal residence. Finally, we constructed three composite medical risk covariate variables consisting of maternal or infant‐related diagnoses that increase the risk of our primary outcomes: maternal comorbid conditions, perinatal complications, and congenital anomalies. Diagnoses which made up these constructed covariates and the specific ICD‐9‐CM codes used to identify them are listed in Table S3.^24^ Models were sequentially adjusted for these medical risk composite covariates, with full models adjusting for all three due to their independent associations with the primary outcomes. All models were clustered by hospital to account for the nonindependence of patients treated at the same hospital.^25^ As a secondary analysis, we clustered by state, which is the level of the treatment (ie, the policy).

We then conducted a series of prespecified stratified analyses. These assessed potential heterogeneous effects on women of different demographic characteristics given previous research, demonstrating the differential effect of parental leave policies on other outcomes by socioeconomic status.^5^, ^13^ Specifically, we assessed for a differential impact by maternal insurance and race/ethnicity by including interaction terms between these variables and the California/post‐PFL interaction variable in fully adjusted models.

Finally, we conducted a series of sensitivity analyses using propensity score matching. Difference‐in‐difference analyses are meant to control for differences that may exist between exposed and unexposed populations. However, given the underlying socioeconomic and racial/ethnic differences that exist between California and the two unexposed states, we created a propensity score based on a mother's likelihood of living in California. Mothers who lived in California in the pre‐PFL period were matched to mothers living in the unexposed states in the pre‐PFL period; mothers who lived in California in the post‐PFL period; and mothers living in the unexposed states in the post‐PFL period. Women were matched on the following factors: race, education, age, insurance coverage, urban vs. rural residence, residence county unemployment rate, presence of any prenatal care, perinatal complications, maternal comorbidities, and presence of congenital anomalies. Conditional logistic regression models adjusting for the same covariates as the difference‐in‐difference models were then conducted for the four outcomes of interest, using two caliper widths: 0.07 and 0.25 standard deviations of the logit odds of the estimated propensity score.

A 2‐sided *P*‐value of <.05 was considered statistically significant for the primary analyses. To avoid the bias of multiple comparisons, Bonferroni correction was used for the stratified analyses. All analyses were conducted in STATA (StataCorp), version 15. This study was deemed exempt by the local institutional review board due to the de‐identified nature of the data.

## 3 RESULTS

### 3.1 Study population

Initially, 6 495 042 births were identified across all three states. After exclusion criteria, there were 5 998 494 births in the nine‐year cohort: 4 323 697 births in California and 1 674 797 births in the unexposed states (1 045 005 births in Pennsylvania and 629 792 births in Missouri). Demographic characteristics that differed across exposed and unexposed states were maternal race/ethnicity and maternal education levels (Table 1). California had fewer non‐Hispanic white mothers and more Hispanic mothers than the other states and more mothers without any high school education.

**TABLE 1:** Maternal and infant characteristics before and after the passage of paid family leave in the exposed (California) and unexposed states (Missouri/Pennsylvania)

	Pre‐PFL (1999‐2003)		Post‐PFL (2005‐2008)	
	Exposed	Unexposed	Exposed	Unexposed
No. of live births	2 319 041	905 438	2 004 656	769 359
Gestational age, weeks (Mean/SD)	38.9 (2.2)	38.8 (1.9)	38.8 (2.2)	38.7 (1.9)
Birthweight, g (mean/SD)	3382 (542)	3355 (568)	3340 (532)	3319 (559)
Male infant (%)	51.2	51.2	51.3	51.3
Cesarean section (%)	24.7	23.5	31.0	30.0
Maternal age (mean/SD)	27.9 (6.3)	27.5 (6.1)	28.0 (6.3)	27.4 (6.0)
Maternal race (%)				
Non‐Hispanic white	35.1	75.5	30.9	74.2
Non‐Hispanic black	5.5	12.5	5.2	14.4
Hispanic	45.8	3.8	49.1	5.0
Asian/Pacific Islander	12.3	1.6	13.6	2.0
Other	1.2	6.7	1.2	4.4
Maternal insurance (%)				
Private insurance	53.5	64.0	48.9	56.5
Public insurance	43.0	31.9	47.9	40.8
Uninsured	0.0	1.6	0.0	1.6
Other	3.5	2.4	3.2	1.1
Maternal education (%)				
No high school	11.9	1.9	10.3	2.1
Some high school	17.6	13.0	20.3	13.2
High school diploma/GED	28.4	33.0	24.9	29.1
Some college	42.1	52.1	44.5	55.7

**FIGURE 1:**
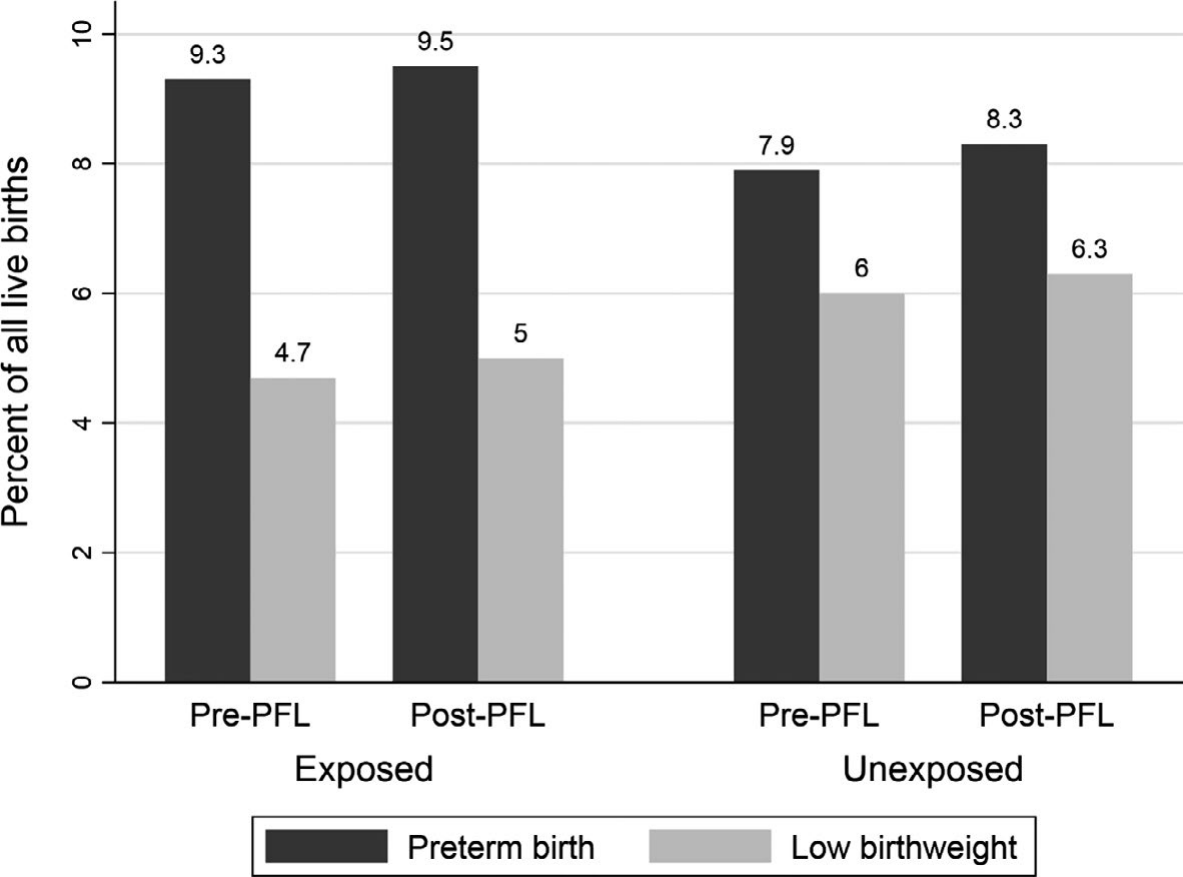
Preterm and low birthweight rates before and after passage of paid family leave in exposed and unexposed states

**FIGURE 2:**
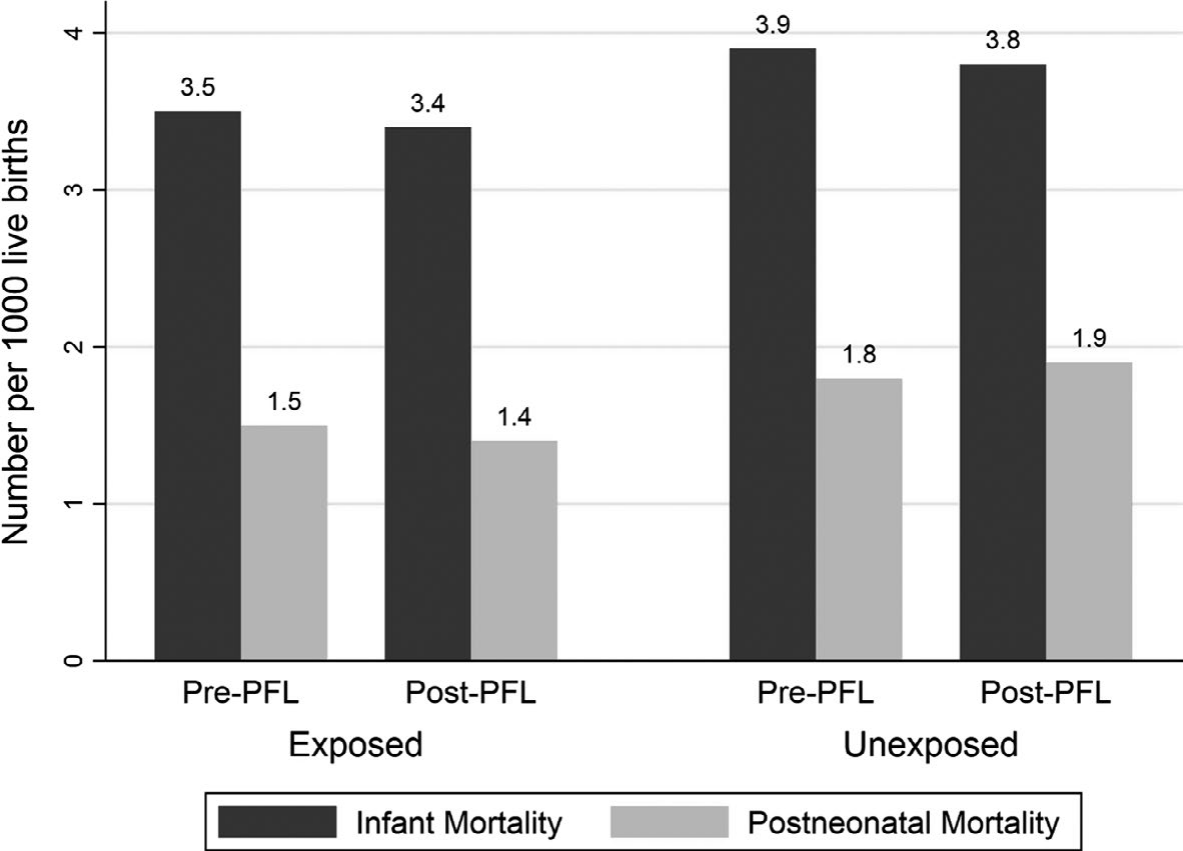
Infant mortality and postneonatal mortality rates before and after passage of paid family leave in exposed and unexposed states

Preterm birth rates increased across study periods in all states as did low birthweight rates (Figure 1). Postneonatal and overall infant mortality rates dropped in California across the study periods, but postneonatal mortality rose in the unexposed states (Figure 2).

### 3.2 Difference‐in‐differences analyses

**FIGURE 3:**
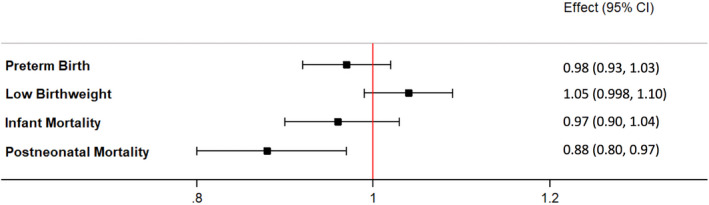
Adjusted odds of outcomes in California compared to unexposed states after 2004 [Colour figure can be viewed at wileyonlinelibrary.com]

In all models, postneonatal mortality was significantly decreased in California after 2004 with Figure 3 showing results of the fully adjusted model (aOR 0.88, 95% CI 0.80‐0.97). This translates into a reduction of 1.94 deaths per 10 000 live births. Differences in the rates of preterm birth, low birthweight, and infant mortality were not statistically significant in the fully adjusted models clustered by delivery hospital. Full details of all models are found in Table S4. When models were clustered by state, the reduced odds of postneonatal mortality were unchanged, but we additionally found lower odds of preterm birth (aOR 0.98, 95% CI 0.97‐0.98) and increased odds of low birthweight (aOR 1.05 95% CI 1.03‐1.06) in California after 2004 (Table S5).

### 3.3 Stratified analyses

Relative to publicly insured women, privately insured women were more likely to have a low birthweight infant (aOR 1.15, 95% CI 1.08‐1.22) in California in the post‐PFL period, even after Bonferroni adjustment. There were no significant interactions between race/ethnicity and the main outcomes in the post‐PFL period. Full details of these models are found in Table 2.

**TABLE 2:** Difference‐in‐differences analyses with estimates stratified by insurance and race/ethnicity

	Preterm birth		Low birthweight		Infant mortality		Postneonatal mortality	
	aOR	95% CI	aOR	95% CI	aOR	95% CI	aOR	95% CI
CA × postpolicy × private insurance	1.00	0.93‐1.07	**1.15**	**1.08‐1.22** *	1.01	0.89‐1.13	1.08	0.92‐1.24
CA × postpolicy × black	1.03	0.92‐1.14	1.05	0.94‐1.16	1.15	0.92‐1.38	1.16	0.91‐1.41
CA × postpolicy × Hispanic	1.01	0.94.1.08	1.01	0.94‐1.08	1.00	0.88‐1.12	1.01	0.83‐1.19
CA × postpolicy × Asian	1.00	0.94‐1.06	1.00	0.93‐1.07	1.01	0.82‐1.20	0.99	0.71‐1.19
CA × postpolicy × Other	0.93	0.75‐1.11	0.95	0.73‐1.17	1.02	0.56‐1.48	0.73	0.24‐1.22

Note

Reference group for insurance interaction was publicly insured women. Reference group for race/ethnicity interactions was non‐Hispanic white women. Models adjusted for time and state fixed effects, maternal age, education, insurance and race, median household income in home census tract, county unemployment rate, congenital anomalies, maternal comorbidities, and perinatal complications. Confidence intervals underwent Bonferroni correction. Models clustered by hospital to adjust for nonindependence of patients treated at the same hospital.

* *P*‐value was < .001.

### 3.4 Sensitivity analyses

Tables S6 and S7 show the standardized differences of our propensity score–matched samples using a 0.07 and 0.25 caliper width. Analyses relied on smaller sample sizes as unmatchable dyads were dropped. The point estimates for the odds ratios for all outcomes were similar to our primary analyses (Table S8), although the confidence intervals were widened reflecting the smaller sample size in the matched samples.

## 4 DISCUSSION

We performed a quasi‐experimental study to assess the impact of the first state‐level paid family leave policy in the United States on several key infant health outcomes. Adjusted postneonatal mortality decreased by 12 percent after the passage of the 2004 Paid Family Leave policy in California. Given the associated effect size of reducing postneonatal deaths by 1.94 per 10,000 live births, we estimate that this translates into 747 fewer infants potentially dying in the first year of life as a result of this policy.

The benefits of paid family leave on infant mortality have been previously well documented in large cohort studies using data from European countries, where there has been widespread adoption of paid family leave at a federal level.^6^, ^8^ This previous literature indicates that *paid* parental leave contributes to fewer low birthweight infants and lower rates of both postneonatal and overall infant mortality.^6^, ^26^ Paid leave has also been associated with lower infant mortality in low‐ to middle‐income countries.^27^ Our work represents the first large‐scale study limited exclusively to US births and the first to find an association between paid parental leave and lower postneonatal mortality in the United States.

These findings align with what we know about the drivers of infant mortality during different periods of infancy. According to the most recent data from the US National Vital Statistics System, the top five causes of infant mortality in this country are congenital malformations, preterm birth/low birthweight, sudden infant death syndrome (SIDS), maternal pregnancy complications, and accidents.^28^ However, postneonatal mortality is a principal driver of the IMR disparity in the United States, and the primary etiologies for postneonatal mortality (such as SIDS and accidents) are highly affected by caregiver behavior.^2^ It is plausible that a family leave program might positively impact caregiver behavior and decrease postneonatal mortality risk factors. A parent who can be home with a secured salary may be able to invest more time in breastfeeding, attend preventive well‐child visits, adequately supervise children, ensure safer sleep environments, or be less stressed overall translating to lower rates of nonaccidental trauma. PFL associations with improved breastfeeding and decreased abusive head trauma have been documented,^13^, ^14^ but the rest of these hypothesized pathways for improved postneonatal mortality rates as a result of PFL must be further investigated. Similarly, the subgroup findings of increased risk of low birthweight among privately insured women merit further study, given that this has not been previously reported. Finally, future studies should also include similar evaluations of subsequently enacted statewide family leave policies since 2004.

We acknowledge some limitations. Administrative and vital statistics data have both advantages and disadvantages. While such datasets allow for the analysis of large cohorts, there is a potential measurement error due to inaccuracies in birth certificate data. In addition, data restrictions did not allow us to assess two important covariates in all three states: marital status and adequacy of prenatal care. As such, these variables could not be included in our adjusted models. Infants whose family moved to another state in the first year of life and then died would have had a death certificate filed in the receiving state, potentially leading to undercounting of deaths in our states of interest. This likely represents a very small percentage of births, but this could not be ascertained in our dataset. We also did not have employment data for individual women; thus, our sample included working and nonworking mothers. However, this means our analyses likely underestimated the effect of the paid family leave policy among employed women. Furthermore, difference‐in‐differences analyses rely on the assumption that the trends seen in the post‐PFL period in Missouri and Pennsylvania are a valid counterfactual for the trends that would have been observed in California had paid family leave not been enacted in 2004. Although we were unable to find any other major changes, events, or policies occurring in the post‐PFL period that might have affected birth outcomes, this cannot be completely excluded. Finally, as other states have continued to enact their own paid family leave policies that at times differ from California's policy with regard to eligibility, maximum benefit, or time provided, the estimated effect we found may not be generalizable to all states.

## 5 CONCLUSIONS

In 2004, California became the first state in the United States to implement a paid family leave (PFL) program. In our study of just under 6 million births among women from three populous states in the United States, California's paid family leave policy was associated with a decreased risk of postneonatal infant mortality. We believe this study offers important insight into our nation's discussion of the social and economic impact of implementing paid family leave policies. It is difficult to fully translate the findings of international studies of paid family leave to the United States, given that the majority of that data originate from countries with longer federally mandated leave or universal health care systems not present here. This study offers evidence of the impact paid family leave can have on postneonatal mortality in the United States, which is the major driver of the overall infant mortality rate disparity we suffer when compared to economically matched peer nations. Given that California has not seen any adverse effects on labor‐/economic‐related outcomes^12^, ^29^, ^30^ and that infant mortality risk differs by geographic region in the United States,^31^ more widespread adoption of this policy may help improve child health nationally.

## CONFLICT OF INTEREST

None of the authors have any conflicts to disclose.

## Supporting information

Author Matrix

Tables S1‐S8
