# Supplementary material for: The impact of paid family leave in the United States on birth outcomes and mortality in the first year of life
Source: Health Serv Res. 2020 Apr 5;55(Suppl 2):807–14. doi: 10.1111/1475-6773.13288 (PMC7518811; doi:10.1111/1475-6773.13288)
Supplement: Supplementary file 2 — Tables S1‐S8 [file HESR-55-807-s002.docx]

**Supplementary Materials**

| **Supplementary Table 1. Models testing the parallel trends assumption necessary for the difference-in-difference approach. There were no significant differences in the trends in the five years prior to 2004.** | | |
| --- | --- | --- |
| **Study Outcomes** | **aOR** | **95% CI** |
| Preterm birth | 0.95 | 0.92-1.00 |
| Low birthweight | 1.01 | 0.97-1.05 |
| Infant mortality | 1.00 | 0.87-1.14 |
| Postneonatal mortality | 0.94 | 0.85-1.04 |

Models adjusted for time and state fixed effects, maternal age, education, insurance and race, median household income in home census tract, county unemployment rate, congenital anomalies, maternal comorbidities and perinatal complications. Models clustered by hospital to adjust for non-independence of patients treated at the same hospital.

| **Supplementary Table 2: Maternal and Infant Characteristics in the year Paid Family Leave was enacted (i.e. 2004)** | | | |
| --- | --- | --- | --- |
|  | **California** | **Missouri** | **Pennsylvania** |
| **No. of live births** | 475283 | 69633 | 118253 |
| **Gestational age, weeks** (Mean/SD) | 38.8 (2.3) | 38.6 (1.9) | 38.7 (2.0) |
| **Birth weight, g** (Mean/SD) | 3354 (546) | 3317 (559) | 3352 (656) |
| **Male infants** (n,%) | 243036 (51.1%) | 35814 (51.4%) | 60575 (51.2%) |
| **Cesareans** (n,%) | 136621 (28.8%) | 19656 (28.2%) | 32916 (27.8%) |
| **Maternal age** (Mean/SD) | 28.1 (6.3) | 26.5 (5.9) | 28.1 (6.1) |
| **Maternal race** (n,%) |  |  |  |
| Non-Hispanic White | 158161 (33.3%) | 53819 (77.3%) | 88447 (74.8%) |
| Non-Hispanic Black | 24047 (5.1%) | 10193 (14.6%) | 15488 (13.1%) |
| Hispanic | 224922 (47.3%) | 3480 (5.0%) | 6373 (5.4%) |
| Other | 68153 (14.3%) | 2141 (3.1%) | 7945 (6.7%) |
| **Maternal insurance** (n,%) |  |  |  |
| Private insurance | 238763 (50.2%) | 35385 (50.8%) | 76533 (64.7%) |
| Public insurance | 220455 (46.4%) | 31334 (45.0%) | 39936 (33.8%) |
| Uninsured | 0 (0.0%) | 1331 (1.9%) | 1251 (1.1%) |
| Other | 16065 (3.4%) | 1583 (2.4%) | 533 (0.5%) |
| **Maternal education** (n,%) |  |  |  |
| No high school | 51050 (10.7%) | 2096 (3.0%) | 1914 (1.6%) |
| Some high school | 79636 (16.8%) | 11023 (15.8%) | 13764 (11.6%) |
| High school diploma/GED | 141398 (29.8%) | 22438 (32.2%) | 33420 (28.3%) |
| Some college | 203199 (42.8%) | 34076 (48.9%) | 69155 (58.5%) |

| **Supplementary Table 3: ICD-9CM Codes for Composite Covariates** | |
| --- | --- |
| **Maternal Comorbid Conditions** | |
| Chronic Hypertension | 642.0x, 642.1x, 642.2x |
| Pregnancy-induced Hypertension | 642.4x, 642.5x, 642.7x |
| Diabetes Mellitus | 250.x, 357.2, 362.0x, 366.41, 648.0x |
| Gestational Diabetes | 648.8x |
| Renal Disease | 646.2x |
| Stroke | 430.0, 431.0, 432.x, 433.x, 433.8x, 433.9x, 434.0x, 434.1x, 434.9x |
| **Perinatal Complications** | |
| Eclampsia | 642.6x |
| Placenta Previa | 641.0x, 641.1x |
| Placenta Abruptio | 641.2x |
| Chorioamnionitis | 658.4x, 659.2x, 659.3x |
| Disorders of Placentation | 641.0x, 641.1x, 641.2x |
| Cord Abnormality | 663.0x, 663.1x, 663.5x |
| Cord Prolapse | 663.0x , 762.4 |
| Eclampsia | 642.6x |
| Oligohydramnios | 658.0x |
| Polyhydramnios | 657.0x |
| Premature Rupture of Membranes | 658.1x, 658.2x |
| **Congenital Anomalies** | |
| Gastrointestinal Malformation | 756.70, 756.79, 750.3, 750.4, 750.5, 750.7, 750.8, 750.9, 751.1, 751.5, 751.8, 751.9, 560.2, 751.4, 751.0, 751.2, 751.3, 771.1, 751.61, 751.7, 751.60, 751.69 |
| Genitourinary Malformation | 753.0, 753.12, 753.14, 753.15, 753.10, 753.19, 753.3, 753.4, 753.21, 753.22, 753.23, 753.29, 753.6, 753.7, 753.8, 753.9, 753.20, 756.71 |
| Central Nervous System Malformation | 741.0x, 741.9x, 742.0, 742.1, 742.2, 742.3, 742.4, 742.59, 742.8, 742.9 |
| Pulmonary Malformation | 519.4, 553.3, 748.9, 750.6, 756.6, 748.3, 748.9, 748.4, 748.60, 748.61, 748.69, 748.8 |
| Cardiac Malformation | 746.3, 746.4, 424.1, 747.10, 747.21, 747.29, 747.11, 747.22, 746.81, 746.7, 425.3, 746.5, 424.0, 746.6, 746.84, 745.10, 745.19, 745.12, 746.85, 425.1, 745.3, 745.11, 745.0, 746.01, 746.83, 746.2, 746.09, 745.2, 746.1, 745.60, 745.61, 745.69, 746.82, 747.41, 747.42, 747.40, 747.49, 746.9, 746.89, 746.87 |
| Skeletal Malformation | 756.50, 756.51, 756.55, 756.56, 756.59 |
| Skin Malformation | 757.1 |
| Chromosomal Anomaly | 758.3, 758.5, 758.89, 758.9, 759.89, 759.9 759.7 759.4 |
| Other Malformation | 778.0, 759.6, 776.5 |

| **Supplementary Table 4. Difference-in-differences estimates of the effect of paid family leave on outcomes in California, with models clustered by hospital to adjust for the non-independence of patients treated at the same hospital.** | | | | | | | | |
| --- | --- | --- | --- | --- | --- | --- | --- | --- |
|  | **Model 1*** | | **Model 2^†^** | | **Model 3^‡^** | | **Model 4^§^** | |
|  | **aOR** | **95 % CI** | **aOR** | **95 % CI** | **aOR** | **95 % CI** | **aOR** | **95 % CI** |
| **Preterm birth** | 0.97 | 0.92-1.02 | 0.98 | 0.93-1.03 | 0.98 | 0.93- 1.03 | 0.98 | 0.93-1.03 |
| **Low birthweight** | 1.03 | 0.99-1.08 | **1.05** | **1.01-1.10** | **1.05** | **1.01-1.10** | 1.05 | 0.998-1.10 |
| **Infant mortality** | 0.98 | 0.91-1.05 | 0.97 | 0.91-1.05 | 0.97 | 0.91-1.04 | 0.97 | 0.90-1.04 |
| **Postneonatal mortality** | **0.88** | **0.80-0.97** | **0.89** | **0.80-0.98** | **0.88** | **0.80-0.97** | **0.88** | **0.80-0.97** |

**Significant findings are bolded.**

*Model 1 includes time and state fixed effects.

**^†^** Model 2 adds adjustment for maternal age, education, insurance, race, obesity, tobacco, alcohol and drug use, median household income in home census tract, county unemployment rate & congenital anomalies.

**^‡^**Model 3 adds adjustment for maternal morbidities.

**^§^**Model 4 adds adjustment for perinatal complications.

| **Supplementary Table 5. Difference-in-differences estimates of the effect of paid family leave on outcomes in California, with models clustered by state.** | | | | | | | | |
| --- | --- | --- | --- | --- | --- | --- | --- | --- |
|  | **Model 1*** | | **Model 2^†^** | | **Model 3^‡^** | | **Model 4^§^** | |
|  | **aOR** | **95 % CI** | **aOR** | **95 % CI** | **aOR** | **95 % CI** | **aOR** | **95 % CI** |
| **Preterm birth** | **0.97** | **0.96-0.98** | **0.98** | **0.98-0.99** | **0.98** | **0.97-0.99** | **0.98** | **0.97-0.98** |
| **Low birthweight** | **1.03** | **1.01-1.05** | **1.05** | **1.02-1.09** | **1.05** | **1.02-1.09** | **1.05** | **1.03-1.06** |
| **Infant mortality** | 0.98 | 0.90-1.07 | 0.97 | 0.91-1.05 | 0.97 | 0.91-1.04 | 0.97 | 0.89-1.05 |
| **Postneonatal mortality** | **0.88** | **0.77-0.99** | **0.89** | **0.81-0.97** | **0.88** | **0.81-0.96** | **0.88** | **0.81-0.96** |

**Significant findings are bolded.**

*Model 1 includes time and state fixed effects.

**^†^** Model 2 adds adjustment for maternal age, education, insurance, race, obesity, tobacco, alcohol and drug use, median household income in home census tract, county unemployment rate & congenital anomalies.

**^‡^**Model 3 adds adjustment for maternal morbidities.

**^§^**Model 4 adds adjustment for perinatal complications.

| **Supplementary Table 6. Propensity Score Variable Matching Using Caliper of 0.07 (N=857,716)** | | | | | |
| --- | --- | --- | --- | --- | --- |
| **Match Variables** | **PRE-CA** | **PRE-PA/MO** | **POST-CA** | **POST-PA/MO** | **Absolute Max Standardized Difference** |
|  | N= 214,429 | N= 214,429 | N= 214,429 | N= 214,429 |  |
| **Race** |  |  |  |  |  |
| White | 0.64 | 0.66 | 0.60 | 0.61 | 0.11 |
| Black | 0.13 | 0.13 | 0.14 | 0.14 | 0.04 |
| Hispanic | 0.13 | 0.12 | 0.16 | 0.15 | 0.13 |
| Other | 0.07 | 0.05 | 0.06 | 0.06 | 0.09 |
| Missing | 0.03 | 0.05 | 0.04 | 0.03 | 0.06 |
| **Education** |  |  |  |  |  |
| Less than HS | 0.04 | 0.03 | 0.03 | 0.04 | 0.06 |
| High School | 0.13 | 0.15 | 0.17 | 0.16 | 0.11 |
| Some college | 0.32 | 0.33 | 0.26 | 0.28 | 0.14 |
| College Degree | 0.51 | 0.50 | 0.54 | 0.52 | 0.09 |
| **Insurance** | 0.61 | 0.61 | 0.55 | 0.53 | 0.17 |
| **Perinatal complications** | 0.21 | 0.21 | 0.20 | 0.22 | 0.03 |
| **Maternal comorbidities** | 0.10 | 0.10 | 0.12 | 0.12 | 0.07 |
| **Congenital anomalies** | 0.01 | 0.02 | 0.02 | 0.02 | 0.01 |
| **Urban vs. rural home** | 0.94 | 0.94 | 0.95 | 0.94 | 0.05 |
| **Unemployment rate** | 5.46 | 5.75 | 5.71 | 5.68 | 0.18 |
| **Prenatal care** | 0.97 | 0.98 | 0.97 | 0.95 | 0.14 |
| **Medically underserved area** | 0.32 | 0.31 | 0.35 | 0.35 | 0.07 |
| **Maternal age** | 27.85 | 27.79 | 27.76 | 27.54 | 0.05 |
| **Propensity Score** | 0.45 | 0.46 | 0.49 | 0.47 | 0.16 |

| **Supplementary Table 7. Propensity Score Variable Matching Using Caliper of 0.25 (N=944,120)** | | | | | |
| --- | --- | --- | --- | --- | --- |
| **Match Variables** | **PRE-CA** | **PRE-PA/MO** | **POST-CA** | **POST-PA/MO** | **Absolute Max Standardized Difference** |
|  | N= 236,030 | N= 236,030 | N= 236,030 | N= 236,030 |  |
| **Race** |  |  |  |  |  |
| White | 0.64 | 0.67 | 0.58 | 0.62 | 0.20 |
| Black | 0.12 | 0.14 | 0.13 | 0.15 | 0.07 |
| Hispanic | 0.13 | 0.10 | 0.19 | 0.14 | 0.24 |
| Other | 0.07 | 0.04 | 0.06 | 0.06 | 0.13 |
| Missing | 0.03 | 0.05 | 0.04 | 0.04 | 0.07 |
| **Education** |  |  |  |  |  |
| Less than HS | 0.04 | 0.03 | 0.03 | 0.04 | 0.04 |
| High School | 0.13 | 0.14 | 0.17 | 0.16 | 0.13 |
| Some college | 0.31 | 0.33 | 0.25 | 0.28 | 0.16 |
| College Degree | 0.53 | 0.50 | 0.54 | 0.52 | 0.08 |
| **Insurance** | 0.62 | 0.62 | 0.55 | 0.53 | 0.19 |
| **Perinatal complications** | 0.20 | 0.21 | 0.19 | 0.22 | 0.06 |
| **Maternal comorbidities** | 0.09 | 0.11 | 0.12 | 0.12 | 0.10 |
| **Congenital anomalies** | 0.01 | 0.02 | 0.01 | 0.02 | 0.02 |
| **Urban vs. rural home** | 0.94 | 0.94 | 0.95 | 0.94 | 0.05 |
| **Unemployment rate** | 5.31 | 5.75 | 5.68 | 5.69 | 0.29 |
| **Prenatal care** | 0.97 | 0.98 | 0.98 | 0.96 | 0.13 |
| **Medically underserved area** | 0.31 | 0.31 | 0.35 | 0.35 | 0.09 |
| **Maternal age** | 27.96 | 27.79 | 27.69 | 27.59 | 0.06 |
| **Propensity Score** | 0.42 | 0.45 | 0.51 | 0.46 | 0.33 |

| **Supplementary Table 8. Adjusted odds of primary outcomes in California after 2004 compared to Missouri/Pennsylvania using propensity score matching** | | |
| --- | --- | --- |
|  | **Adjusted Odds Ratio** | **95% Confidence Interval** |
| **0.07 Caliper** |  |  |
| Preterm Birth | 0.99 | 0.95-1.02 |
| Low birthweight | 1.04 | 0.99-1.09 |
| Infant mortality | 1.01 | 0.84-1.21 |
| Postneonatal mortality | 0.83 | 0.64-1.07 |
| **0.25 Caliper** |  |  |
| Preterm Birth | 0.97 | 0.93-1.00 |
| Low birthweight | **1.05** | **1.00-1.10** |
| Infant mortality | 0.96 | 0.80-1.14 |
| Postneonatal mortality | 0.85 | 0.66-1.10 |
